# P-2189. Performance of Dried Blood Spots Cards for Serologic Detection of HPV16 Antibodies in Oropharyngeal Squamous Cell Carcinoma Patients

**DOI:** 10.1093/ofid/ofaf695.2352

**Published:** 2026-01-11

**Authors:** Maisha Maliha Rahman, Soma Bose, Li Chen, Jessica Burris, Rony Aouad, Susanne Arnold, Melvyn Yeoh, Birgitta Michels, Tim Waterboer, Krystle Lang Kuhs

**Affiliations:** University of Kentucky, Lexington, KY; University of Kentucky, Lexington, KY; University of Kentucky, Lexington, KY; University of Kentucky, Lexington, KY; University of Kentucky, Lexington, KY; University of Kentucky, Lexington, KY; University of Kentucky, Lexington, KY; German Cancer Research Center (DKFZ), Heidelberg, Baden-Wurttemberg, Germany; German Cancer Research Center (DKFZ), Heidelberg, Baden-Wurttemberg, Germany; University of Kentucky, Lexington, KY

## Abstract

**Background:**

Antibodies against the human papillomavirus type 16 (HPV16) E6 oncoprotein are a promising biomarker for the early detection of HPV-driven oropharyngeal squamous cell carcinoma (HPV+OPSCC). However, standard serologic testing poses logistical barriers in underserved regions where HPV+OPSCC incidence is high. Dried blood spot (DBS) cards offer a low-resource alternative, but have not been evaluated for HPV antibody detection.

**Methods:**

25 OPSCC patients were recruited from the University of Kentucky and provided paired serum (venipuncture) and DBS (finger prick) samples. HPV16 antibodies (L1, E1, E2, E4, E6, E7) were measured using multiplex serology; levels were quantified as median fluorescence intensity (MFI) and dichotomized using established cutoffs. Correlation between serum (gold standard) and DBS MFI values was assessed using linear regression and Bland-Altman plots. Sensitivity, specificity, and Cohen’s kappa were calculated to evaluate agreement.

**Results:**

Mean MFI levels were lower in DBS than serum, but values were strongly correlated with R² ranging from 0.54 to 0.93. The R-squared for HPV16 E6 antibodies was 0.68. Among the 20 HPV16 E6 seropositive participants (serum), 18 were seropositive on DBS (Sensitivity: 90%); all five HPV16 E6 seronegative participants (serum) also tested seronegative on DBS (Specificity: 100%; kappa: 0.787). Specificity was 100% across all markers, while sensitivity varied from 0% (HPV16 L1) to 100% (HPV16 E2).

**Conclusion:**

DBS cards are an accurate, inexpensive, and scalable alternative to serum-based testing for HPV16 E6 antibodies, particularly in medically underserved regions. Further studies are needed to validate these findings and support broader implementation.

**Disclosures:**

All Authors: No reported disclosures

